# Supplementary material for: Inferring spatial transcriptomics markers from whole slide images to characterize metastasis-related spatial heterogeneity of colorectal tumors: A pilot study
Source: J Pathol Inform. 2023 Mar 29;14:100308. doi: 10.1016/j.jpi.2023.100308 (PMC10127126; doi:10.1016/j.jpi.2023.100308)
Supplement: Supplementary file 1 — Supplementary material [file mmc1.docx]

**Appendix**

**Supplementary Table 1.** Comparison of model performance for predicting dichotomized and log-transformed expression for genes across the whole transcriptome. These statistics are created by taking the median across all genes, reported for each held-out slide.

|  |  | Classification | | Regression | |
| --- | --- | --- | --- | --- | --- |
| Model | **Slide** | **AUROC** | **AP** | **Spearman R** | **Pearson R** |
| Inception-256 | Overall | 0.768 | 0.603 | 0.444 | 0.477 |
|  | A1 | 0.78 | 0.675 | 0.494 | 0.511 |
|  | B1 | 0.834 | 0.796 | 0.612 | 0.605 |
|  | C1 | 0.625 | 0.432 | 0.214 | 0.301 |
|  | D1 | 0.831 | 0.51 | 0.454 | 0.491 |
| Inception-512 | Overall | 0.781 | 0.63 | 0.461 | 0.498 |
|  | A1 | 0.79 | 0.688 | 0.515 | 0.537 |
|  | B1 | 0.856 | 0.816 | 0.639 | 0.647 |
|  | C1 | 0.629 | 0.459 | 0.22 | 0.299 |
|  | D1 | 0.847 | 0.555 | 0.469 | 0.51 |
| Inception-768 | Overall | 0.787 | 0.639 | 0.486 | 0.54 |
|  | A1 | 0.802 | 0.705 | 0.551 | 0.578 |
|  | B1 | 0.864 | 0.823 | 0.671 | 0.655 |
|  | C1 | 0.636 | 0.474 | 0.239 | 0.4 |
|  | D1 | 0.845 | 0.553 | 0.481 | 0.525 |
| GAT-1 | Overall | 0.743 | 0.539 | 0.413 | 0.43 |
|  | A1 | 0.785 | 0.669 | 0.364 | 0.376 |
|  | B1 | 0.811 | 0.73 | 0.603 | 0.582 |
|  | C1 | 0.606 | 0.409 | 0.239 | 0.335 |
|  | D1 | 0.77 | 0.349 | 0.444 | 0.428 |
| GAT-2 | Overall | 0.677 | 0.444 | 0.407 | 0.45 |
|  | A1 | 0.769 | 0.645 | 0.511 | 0.545 |
|  | B1 | 0.772 | 0.612 | 0.574 | 0.568 |
|  | C1 | 0.589 | 0.333 | 0.146 | 0.265 |
|  | D1 | 0.578 | 0.184 | 0.421 | 0.428 |
| GAT-4 | Overall | 0.702 | 0.462 | 0.453 | 0.499 |
|  | A1 | 0.679 | 0.571 | 0.517 | 0.551 |
|  | B1 | 0.764 | 0.614 | 0.622 | 0.643 |
|  | C1 | 0.604 | 0.379 | 0.228 | 0.356 |
|  | D1 | 0.759 | 0.283 | 0.445 | 0.454 |
| GAT-4-AXIN2 | Overall | 0.702 | 0.462 | 0.545 | 0.578 |
|  | A1 | 0.679 | 0.57 | 0.511 | 0.446 |
|  | B1 | 0.764 | 0.614 | 0.745 | 0.771 |
|  | C1 | 0.604 | 0.379 | 0.286 | 0.46 |
|  | D1 | 0.759 | 0.283 | 0.637 | 0.633 |
| GAT-4-CDH1 | Overall | 0.800 | 0.707 | 0.546 | 0.571 |
|  | A1 | 0.826 | 0.747 | 0.468 | 0.478 |
|  | B1 | 0.858 | 0.791 | 0.753 | 0.741 |
|  | C1 | 0.648 | 0.462 | 0.31 | 0.425 |
|  | D1 | 0.867 | 0.827 | 0.652 | 0.64 |
| GAT-1-ZINB | Overall | n/a | n/a | 0.393 | 0.408 |
|  | A1 | n/a | n/a | 0.441 | 0.429 |
|  | B1 | n/a | n/a | 0.551 | 0.562 |
|  | C1 | n/a | n/a | 0.15 | 0.19 |
|  | D1 | n/a | n/a | 0.429 | 0.449 |
| GAT-4-ZINB | Overall | n/a | n/a | 0.41 | 0.432 |
|  | A1 | n/a | n/a | 0.485 | 0.542 |
|  | B1 | n/a | n/a | 0.602 | 0.621 |
|  | C1 | n/a | n/a | 0.128 | 0.2 |
|  | D1 | n/a | n/a | 0.426 | 0.364 |
| ViT-224 | Overall | 0.756 | 0.583 | 0.436 | 0.454 |
|  | A1 | 0.768 | 0.644 | 0.511 | 0.506 |
|  | B1 | 0.826 | 0.762 | 0.601 | 0.567 |
|  | C1 | 0.604 | 0.413 | 0.177 | 0.255 |
|  | D1 | 0.827 | 0.513 | 0.455 | 0.487 |
| ViT-384 | Overall | 0.765 | 0.601 | 0.446 | 0.468 |
|  | A1 | 0.794 | 0.678 | 0.513 | 0.521 |
|  | B1 | 0.836 | 0.788 | 0.612 | 0.583 |
|  | C1 | 0.606 | 0.406 | 0.213 | 0.307 |
|  | D1 | 0.823 | 0.530 | 0.446 | 0.462 |

**
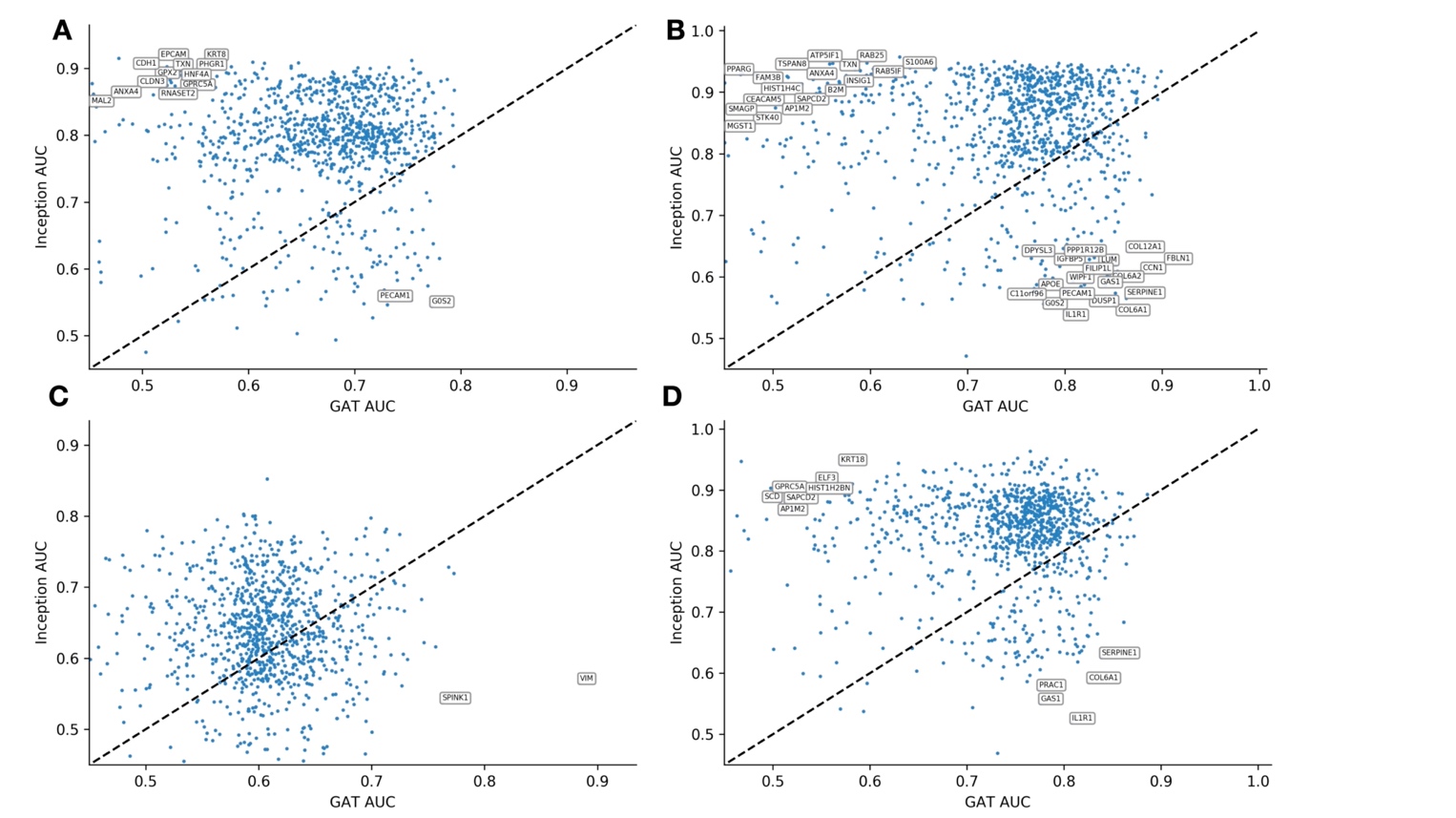
**

**Supplementary Figure 1: Scatterplot depicting gene-specific AUCs for GAT and Inception-768 for slides: A)** A1; **B)** B1; **C)** C1; **D)** D1

**
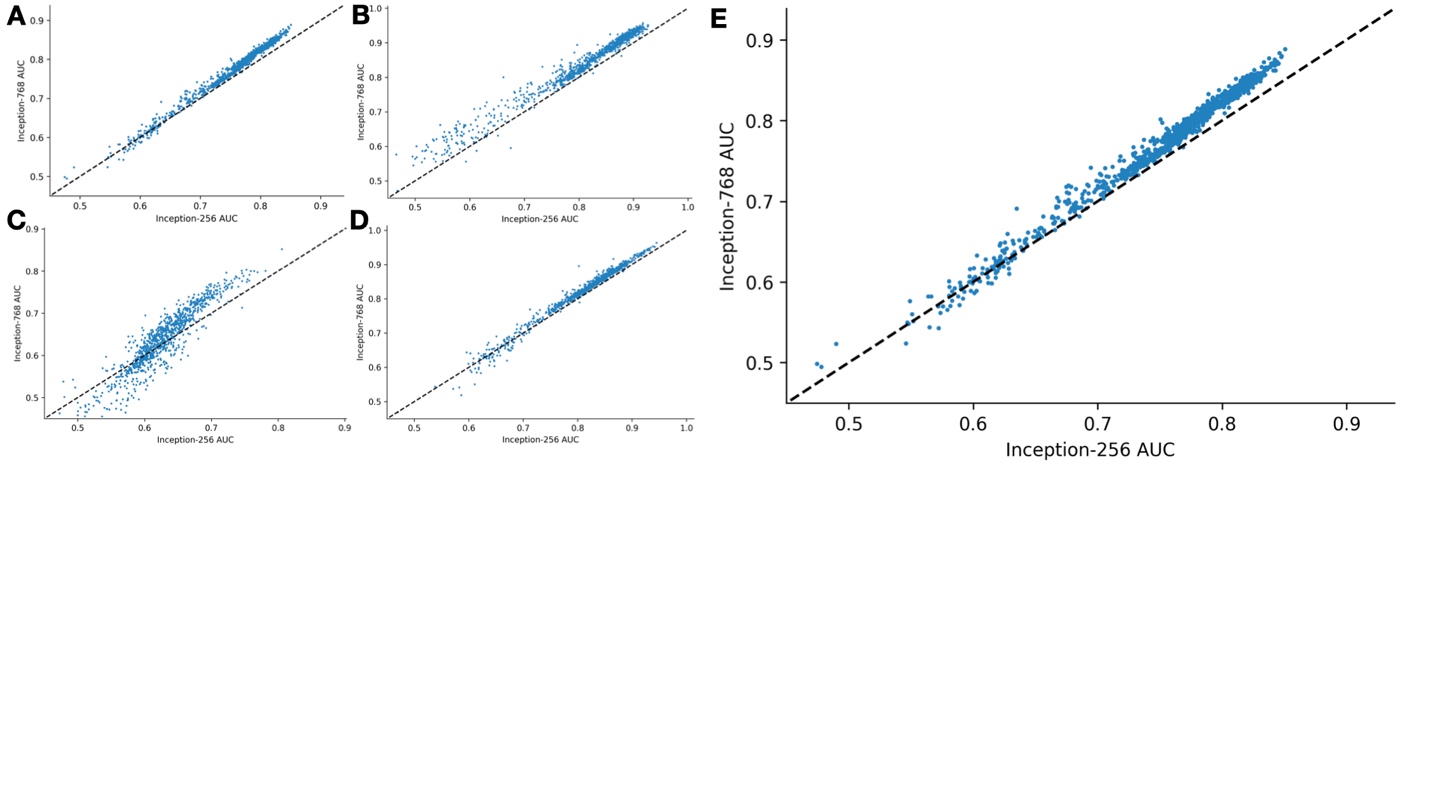
**

**Supplementary Figure 2: Scatterplot depicting gene-specific AUCs for Inception-256 and Inception-768 for slides: A)** A1; **B)** B1; **C)** C1; **D)** D1, **E)** Overall

**Supplementary Table 2: Enrichr pathway results for genes found to be accurately predicted from the tissue histology via the Inception and GAT approaches;** pathways were filtered based on tissue specificity

|  | **Inception** | | |  | **GAT** | | |
| --- | --- | --- | --- | --- | --- | --- | --- |
| **Term** | | **Overlap** | **Adjusted P-value** | **Term** | | **Overlap** | **Adjusted P-value** |
| EPCAM in Cancer Cell Motility and Proliferation | | 9/36 | 2.79e-07 | Proteins with Altered Expression in Cancer Metastases | | 9/106 | 1.22e-03 |
| Proteins with Altered Expression in Cancer Metastases | | 11/106 | 2.82e-05 | Desmosome Assembly | | 4/18 | 4.56e-03 |
| Epithelial Cell in the Saliva Formation | | 5/13 | 7.15e-05 | Corneodesmosomes in Atopic Dermatitis | | 4/19 | 5.15e-03 |
| Androgens in Sebocyte Maturation | | 8/65 | 1.69e-04 | Proteins with Altered Expression in Cancer Metabolic Reprogramming | | 7/85 | 5.50e-03 |
| Proteins Involved in Ulcerative Colitis | | 11/141 | 1.69e-04 | WNT in Epithelial to Mesenchymal Transition in Cancer | | 5/43 | 8.09e-03 |
| Desmosome Assembly | | 5/18 | 1.70e-04 | Metabolic Effects of Oncogenes and Tumor Suppressor in Cancer Cells | | 6/68 | 8.09e-03 |
| Corneodesmosomes in Atopic Dermatitis | | 5/19 | 2.03e-04 | Proteins Involved in Myocardial Ischemia | | 11/252 | 1.18e-02 |
| Proteins Involved in Colorectal Neoplasms | | 9/99 | 2.35e-04 | Desmosome Dysfunction in Cardiomyocyte | | 3/12 | 1.26e-02 |
| WNT in Epithelial to Mesenchymal Transition in Cancer | | 6/43 | 5.66e-04 | RAGE/AGER and S100 Proteins in Cardiovascular Injury | | 4/30 | 1.56e-02 |
| Acinar Cells in the Saliva Formation | | 4/13 | 5.66e-04 | Desmosomes Role in Dilated Cardiomyopathy | | 3/15 | 2.31e-02 |
| Epithelial to Mesenchymal Transition in Cancer: Overview | | 8/90 | 5.85e-04 | Epithelial to Mesenchymal Transition in Cancer: Overview | | 6/90 | 2.58e-02 |
| Metastatic Colorectal Cancer | | 9/121 | 7.01e-04 | Proteins Involved in Atherosclerosis | | 9/200 | 2.58e-02 |
| Proteins with Altered Expression in Cancer Metabolic Reprogramming | | 7/85 | 2.43e-03 | Cetuximab Resistance in Colorectal Cancer | | 5/64 | 3.05e-02 |
| Telogen Maintenance in Androgenic Alopecia | | 4/23 | 4.15e-03 | Proteins Involved in Colorectal Neoplasms | | 6/99 | 3.41e-02 |
| Cancer Cells Inhibit Adipocyte Differentiation | | 4/27 | 6.43e-03 | Glycolysis Activation in Cancer (Warburg Effect) | | 4/40 | 3.41e-02 |
| TGFB Family in Epithelial to Mesenchymal Transition in Cancer | | 6/80 | 8.55e-03 |  |  | | |
| Fatty Acid Synthase (FASN) Signaling | | 3/16 | 1.52e-02 |  |  |  |  |
| WNT Signaling Activation by Blocking of Tumor Suppressors | | 4/36 | 1.57e-02 |  |  |  |  |
| HPV Infection and Cancer | | 5/65 | 1.80e-02 |  |  |  |  |
| Androgens in Adipocyte Activation | | 3/18 | 1.80e-02 |  |  |  |  |
| Glycolysis Activation in Cancer (Warburg Effect) | | 4/40 | 1.97e-02 |  |  |  |  |
| Metabolic Effects of Oncogenes and Tumor Suppressor in Cancer Cells | | 5/68 | 1.98e-02 |  |  |  |  |
| Proteins Involved in HPV Infection | | 3/19 | 1.98e-02 |  |  |  |  |
| CDH1 Down regulation Promotes Cancer Cell Migration and Metastases | | 4/44 | 2.45e-02 |  |  |  |  |
| Androgen Deficiency in Male Obesity | | 3/24 | 3.31e-02 |  |  |  |  |
| Sialophorin -> CTNNB/MYC/TP53 Signaling | | 3/25 | 3.60e-02 |  |  |  |  |
| mRNA Degradation | | 3/26 | 3.91e-02 |  |  |  |  |
| WNT Canonical Signaling Activation in Cancer | | 3/27 | 4.16e-02 |  |  |  |  |
| Proteins Involved in Arterial Hypertension | | 9/255 | 4.30e-02 |  |  |  |  |
| Hyaluronic Acid, CD44 and HMMR in Cancer Cell Invasion and Survival | | 4/56 | 4.35e-02 |  |  |  |  |
| Estrogen Deficiency in Female Obesity | | 2/9 | 4.35e-02 |  |  |  |  |
| Adipocyte Hypertrophy and Hyperplasia | | 3/29 | 4.50e-02 |  |  |  |  |
| Proteins Involved in Hepatocellular Carcinoma | | 6/130 | 4.74e-02 |  |  |  |  |
| Adherens Junction Assembly (Cadherins) | | 3/30 | 4.83e-02 |  |  |  |  |
| Proteins with Altered Expression in Cancer-Associated Sustaining of Proliferative Signaling | | 7/175 | 5.00e-02 |  |  |  |  |

**
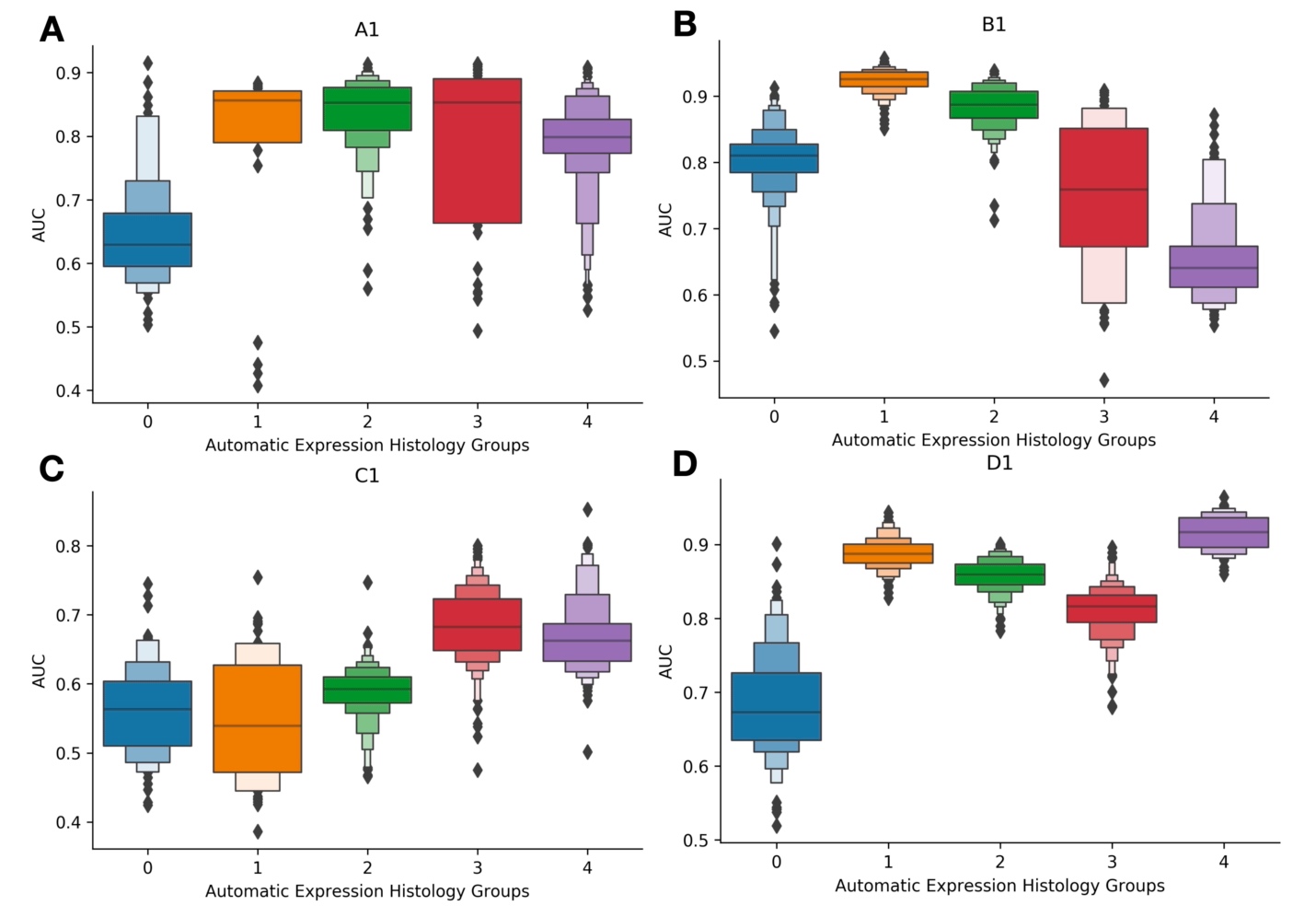
**

**Supplementary Figure 3: Boxenplots illustrating the predictive accuracy of Inceptionv3 (AUC, y-axis) across genes,** **separated by the genes’ Automatic Expression Histology groups (colors, x-axis)**; gathered from validation slides held-out of the training/validation set. Different groups were assigned for each slide. **A-D)** correspond to slides A1-D1 respectively

**
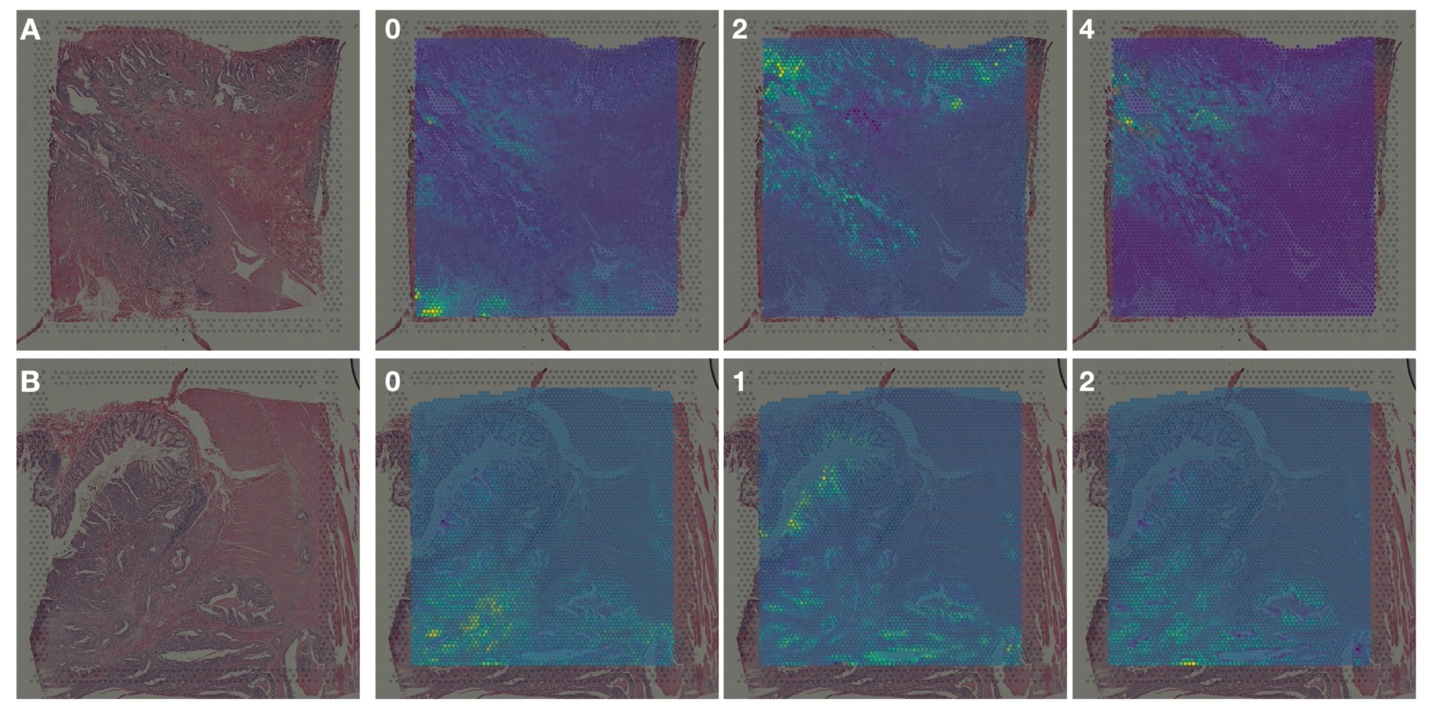
**

**Supplementary Figure 4: Spatial expression patterns for select AEH groups for slides: A)** A1; **B)** B1; genes from the first featured AEH group for each slide were predicted with low accuracy; genes from the final two AEH groups were predicted with high accuracy

**
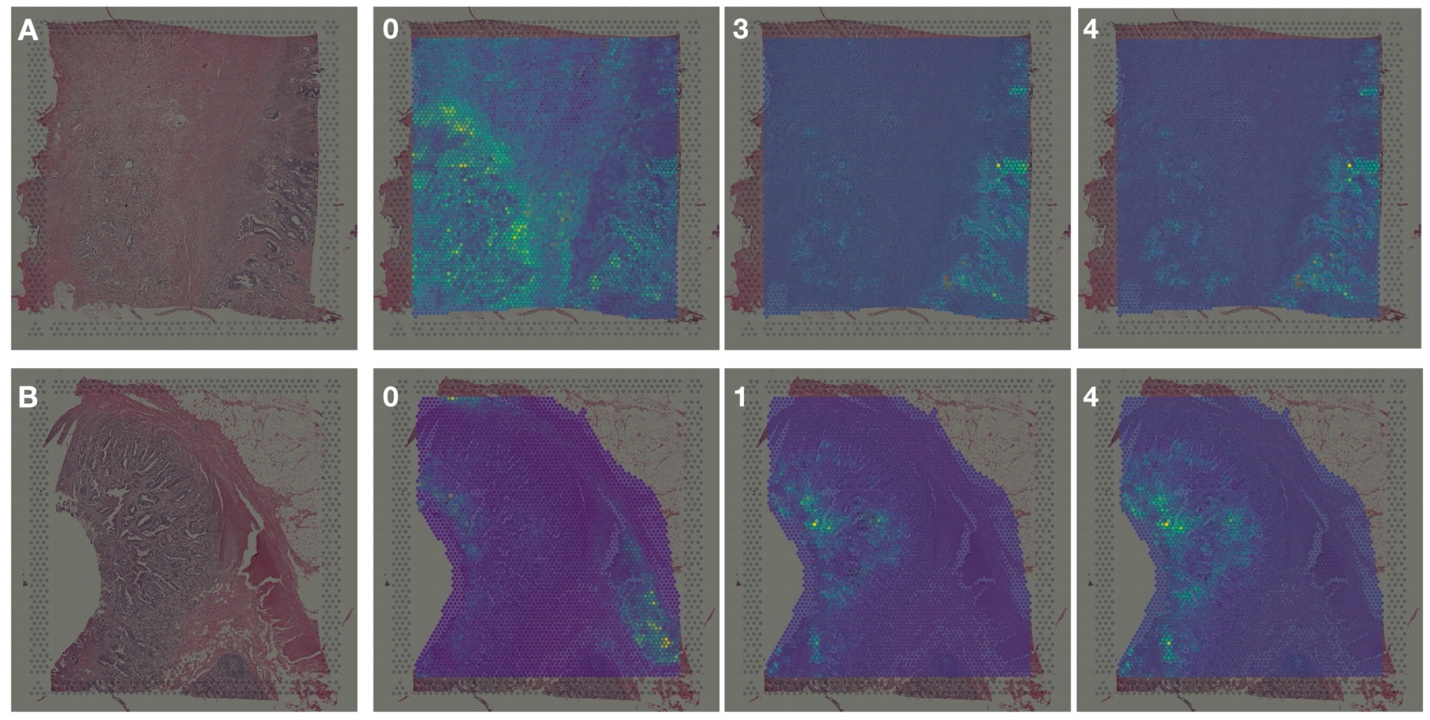
**

**Supplementary Figure 5: Spatial expression patterns for select AEH groups for slides: A)** C1; **B)** D1; genes from the first featured AEH group for each slide were predicted with low accuracy; genes from the final two AEH groups were predicted with high accuracy

**Supplementary Table 3: Select genes differentially spatially autocorrelated between patients with and without concurrent metastasis and associated pathways;** determined using enrichr

| Ground Truth | | | |
| --- | --- | --- | --- |
| Randomly Selected Genes | **Pathways** | **Overlap** | **Adj. P-Value** |
| STXBP3 | Epithelial Mesenchymal Transition | 17/200 | 2.53e-09 |
| IMPAD1 | Coagulation | 11/138 | 0.000006432 |
| ING1 | TGF-beta Signaling | 11/200 | 0.002024 |
| YY1AP1 | Myogenesis | 11/200 | 0.0001214 |
| IGFBP4 | Apical Junction | 10/200 | 0.0001214 |
| ACO1 | Hypoxia | 5/54 | 0.0005478 |
| ID3 | Cholesterol Homeostasis | 6/144 | 0.02979 |
| PTAR1 | UV Response Dn | 7/199 | 0.02582 |
| SYNM | UV Response Up | 7/200 | 0.0286 |
| SPRY2 | IL-2/STAT5 Signaling | 7/200 | 0.02582 |
| RABL3 | Estrogen Response Late | 6/158 | 0.02582 |
| N4BP2L2 | p53 Pathway | 4/74 | 0.02582 |
| Inception | | | |
| Randomly Selected Genes | **Pathways** | **Overlap** | **Adj. P-Value** |
| DPYSL3 | Epithelial Mesenchymal Transition | 9/200 | 3.41e-13 |
| CALD1 | TGF-beta Signaling | 3/138 | 0.004267 |
| G0S2 | Coagulation | 3/200 | 0.001833 |
| IGHG1 | TNF-alpha Signaling via NF-kB | 2/54 | 0.003624 |
| LIMS2 | Angiogenesis | 2/200 | 0.05432 |
| COL6A2 | Xenobiotic Metabolism | 2/200 | 0.02452 |
| C1S | Hypoxia | 2/200 | 0.02452 |
| SERPINF1 | Myogenesis | 2/200 | 0.02452 |
| FOSB | Complement | 2/200 | 0.02452 |
| SORBS1 | Inflammatory Response | 1/36 | 0.02452 |
| WIPF1 | IL-6/JAK/STAT3 Signaling | 1/87 | 0.1169 |
| PCOLCE | Interferon Alpha Response | 1/97 | 0.119 |
| GAT | | | |
| Randomly Selected Genes | **Pathways** | **Overlap** | **Adj. P-Value** |
| ACTA2 | Epithelial Mesenchymal Transition | 18/200 | 1.85e-27 |
| COL5A2 | Angiogenesis | 4/36 | 0.000002955 |
| COL3A1 | Myogenesis | 5/200 | 0.00009592 |
| COL12A1 | Glycolysis | 3/200 | 0.01585 |
| GREM1 | Apical Junction | 3/200 | 0.01585 |
| CDH11 | Hedgehog Signaling | 2/138 | 0.1196 |
| SPARCL1 | Apical Surface | 2/144 | 0.1321 |
| CRYAB | Coagulation | 2/200 | 0.06564 |
| POSTN | UV Response Dn | 2/200 | 0.06564 |
| TPM2 | TGF-beta Signaling | 1/36 | 0.1475 |
| DES | Hypoxia | 1/44 | 0.09354 |
| DCN | Adipogenesis | 1/54 | 0.09354 |
